# Structural changes in gray matter and white matter across the Alzheimer’s disease continuum

**DOI:** 10.3389/fnins.2026.1853132

**Published:** 2026-08-05

**Authors:** Shuai Lin, Ming Xue, Tianqi Wang, Yue Chen, Chenjun Sheng, Jiali Sun, Ying Sui, Wenxuan Zou, Jianxiu Lian, Jing Peng, Tong Zhang, Wei Wang

**Affiliations:** 1 Department of MRI, First Affiliated Hospital of Harbin Medical University, Harbin, Heilongjiang Province, China; 2 Department of Radiology, First Affiliated Hospital of Harbin Medical University, Harbin, Heilongjiang Province, China; 3 Philips Healthcare, Beijing, China; 4 Department of Radiology, The Fourth Affiliated Hospital of Harbin Medical University, Harbin, Heilongjiang Province, China

**Keywords:** Alzheimer’s disease, gray matter, magnetic resonance imaging, neurodegeneration, white matter

## Abstract

**Objective:**

The pathophysiological spectrum of Alzheimer’s disease (AD) is a continuum, with the AT(N) framework defining stages based on the presence of Aβ pathology, tau pathology, and neurodegeneration. However, the spatial characteristics of gray matter (GM) and white matter (WM) changes at different stages remain insufficiently defined. This study aimed to establish a cross-sectional cohort of the AD continuum using the AT(N) staging system and systematically delineate the spatial features of GM and WM changes across this spectrum.

**Methods:**

This study included subjects across the AD continuum with defined AT(N) stages [*n* = 178, including A-T- cognitive normal (CN), A + T- CN, A + T + CN, A + T + mild cognitive impairment (MCI), and A + T + AD subgroups]. Voxel-based morphometry (VBM) was used to assess GM volume changes. Tract-based spatial statistics (TBSS) was employed to analyze WM microstructure alterations using fractional anisotropy (FA). Group comparisons were performed to characterize the pattern of brain structural alterations across different AT(N) stages. Correlation analyses were performed to assess correlations of GM and WM changes with AD neuropathology and with cognitive function, respectively.

**Results:**

A region-specific pattern of GM volume atrophy was observed: hippocampal (HIP) volume atrophy was most pronounced in the A + T- CN subgroup. The parahippocampal gyrus (PHG), amygdala (AMYG), and fusiform gyrus (FFG) showed volume atrophy in the A + T + CN subgroup. In the A + T + MCI and A + T + AD subgroups, extensive GM volume reduction involved global GM regions. For WM alterations, reduced FA values were observed in the hippocampal cingulum (CgH) at the A + T + CN stage. In A + T + MCI and A + T + AD subgroups, a broader scope of WM FA reduction was observed. Correlation analyses revealed that HIP volume was positively associated with CgH FA values and Aβ levels, while both HIP volume and CgH FA values were negatively associated with P-tau levels, and both HIP volume and CgH FA values positively correlated with global cognition and memory performance.

**Conclusion:**

GM and WM alterations are widespread in the AD continuum. Early selective GM volume loss and impaired WM axonal integrity are associated with pathological processes in AD.

## 1 Introduction

The global prevalence of dementia was estimated at 57 million individuals in 2019, with projections indicating an increase to 153 million by the year 2050 (GBD 2019 Dementia Forecasting Collaborators, 2022). Alzheimer’s disease (AD), the most prevalent form of dementia, is characterized by progressive memory loss and cognitive impairment (Livingston et al., 2024). Notably, pathological changes in the brain associated with AD have been found to commence approximately 10 to 20 years prior to the manifestation of clinical symptoms, accumulating and exacerbating in a specific temporal and spatiotemporal sequence (Jia et al., 2024; Therriault et al., 2024). The initial pathological process involves the extracellular deposition of β-amyloid (Aβ), leading to the formation of senile plaques. This accumulation subsequently triggers hyperphosphorylation of tau protein and neuroinflammation (Liu et al., 2024). Hyperphosphorylated tau (P-tau) exhibits reduced affinity for microtubules, resulting in the formation of neurofibrillary tangles (Gonzalez-Ortiz et al., 2023; Karikari et al., 2022). Furthermore, evidence suggests that P-tau propagates between neurons via extracellular pathways (Bloom, 2014). These pathological processes lead to extensive neuronal damage and are associated with disease severity. Consequently, P-tau serves as a critical biomarker for pathological staging of AD.

The National Institute on Aging and the Alzheimer’s Association (NIA-AA) proposed the AT(N) framework, defining AD as a continuous disease spectrum based on the presence or absence of Aβ deposition (A), pathological tau (T), and neurodegeneration (N) biomarkers (Jack et al., 2018). Aβ deposition (A) and pathological tau (T) are regarded as specific indicators of AD, whereas neurodegeneration (N) biomarkers reflect downstream neurodegenerative changes that may arise from various etiologies (Li et al., 2020). Therefore, using the AT(N) framework together with cognitive status provides a reliable model for in vivo MRI analysis of AD pathology and cognition.

The initial brain regions affected in AD are the medial temporal lobe structures, specifically the entorhinal cortex and hippocampus (HIP). These areas possess extensive white matter (WM) fiber connections with distant cortical regions (Chen Y. et al., 2023). Subsequently, involvement extends to the frontal, parietal, and temporal lobes (Crane et al., 2024). This pattern of brain structural changes in the AD continuum follows the Braak staging framework (Braak and Braak, 1991). It has been concluded that there is heterogeneity in the extent of gray matter (GM) and WM changes in different stages of AD. Quantifying and elucidating the differences in GM and WM changes at various stages of AD will enhance our understanding of the individualized progression trajectory of AD and clarify the synergistic effects between GM atrophy and WM alterations.

In this study, the structural alterations patterns of GM and WM were studied using voxel-based morphometry (VBM) and tract-based spatial statistics (TBSS). This research aimed to elucidate the patterns of brain structural alterations and their association with pathological markers, especially to reveal the differences of GM and WM alterations in different AD pathological states and cognitive levels. The findings will provide critical imaging evidence to support early diagnosis, biomarker identification, and personalized intervention strategies for AD.

## 2 Methods

### 2.1 Subjects

All subjects were recruited from the ADNI database (https://adni.loni.usc.edu/), the project conforms to the medical ethics, and each involved research unit is approved by the local ethical review committee. Informed consent was obtained from all participants. A total of 178 subjects were enrolled from the ADNI database, including 97 cognitive normal (CN) subjects, 39 mild cognitive impairment (MCI) patients, and 42 AD patients. All subjects underwent 3D T1-weighted imaging (T1WI) and diffusion tensor imaging (DTI) examinations, APOE ε4 genotype detection, cerebrospinal fluid (CSF) marker and neuropsychological scales assessment. All subjects were diagnosed according to the criteria of the National Institute of Neurological and Communicative Disorders and Stroke-Alzheimer’s Disease and Related Disorders Association (NINCDS-ADRDA) (McKhann et al., 2011).

Demographic information included age, sex, and years of education. Neuropsychological scales included Mini-mental State Examination (MMSE), Montreal Cognitive Assessment (MOCA), Wechsler Memory Scale Logical Memory (WMS-LM), Clinical Dementia Rating (CDR), Alzheimer’s Disease Assessment Scale (ADAS: ADAS-Cog11, ADAS-Cog13, ADAS-Q4) and Rey Auditory-Verbal Learning Test (RAVLT: RAVLT immediate, RAVLT learning, RAVLT forgetting, and RAVLT percent forgetting). Based on prior research establishing cognitive composites for cognitive impairment studies (Long et al., 2026; Qiu et al., 2025; Wan et al., 2021; Wang et al., 2018), the global cognition composite was derived from the MMSE, MoCA, and ADAS-Cog13, and the memory composite was derived from WMS-LM, RAVLT immediate, RAVLT learning, and RAVLT percent forgetting. Details were provided in Supplementary material S1.

According to the AT(N) staging criteria, patients were categorized into five groups: Group 1: A-T- CN (*n* = 30); Group 2: A + T- CN (*n* = 40); Group 3: A + T + CN (*n* = 27); Group 4: A + T + MCI (*n* = 39); and Group 5: A + T + AD (*n* = 42). The criterion for A was defined as Aβ_1-42_ in CSF, with a threshold of < 880 pg./mL indicating a positive result. For the T marker, the level of P-tau_181_ in CSF > 19.2 pg./mL was considered indicative of a positive result (Hansson et al., 2018; Zeng et al., 2022). The N biomarker was excluded from the grouping criteria, as the GM atrophy and WM microstructural alterations evaluated in this study constitute established N biomarkers for neurodegeneration and were designated as the primary outcome measures rather than grouping criteria (Li et al., 2020).

### 2.2 Image acquisition

All imaging data used in this study were obtained from a 3 T MRI scanner. 3D T1WI specific sequence parameters are as follows: (1) GE: TR/TE = 7.7–6.9/3.1–2.8 ms, TI = 400 ms, layer thickness = 1.2 mm, matrix = 256 × 256 × 196; (2) Philips: TR/TE = 6.8/3.2 ms, flip angle = 9°, layer thickness = 1.2 mm, matrix = 256 × 256 × 170; (3) Siemens: TR/TE = 2300/3.0 ms, TI = 900 ms, layer thickness = 1.2 mm, matrix = 240 × 256 × 176. DTI specific sequence parameters are as follows: (1) GE: TR/TE = 7800/55–62 ms; flip angle = 90°; field of view = 232 × 232 × 138 mm^3^; (2) Siemens: TR/TE = 7,200–9,600/56–82 ms; flip angle = 90°; field of view = 232 × 232 × 160 mm^3^; (3) Philips: TR/TE = 9000/59 ms, flip angle = 90°; field of view = 232 × 200 × 125 mm^3^. 6 images without diffusion-weighted (*b* = 0 s/mm^2^) and 48 images with diffusion-weighted (*b* = 1,000 s/mm^2^). More information can be seen on the ADNI scanning website (http://adni.loni.usc.edu/methods/mri-tool/mri-analysis/).

### 2.3 Image post-processing

SPM12 software (https://www.fil.ion.ucl.ac.uk/spm/software/spm12) and the CAT12 toolkit (r2170 version, https://neuro-jena.github.io/cat12-help/) were used for 3D T1WI image preprocessing and segmentation. The specific steps were as follows: (1) Skull stripping; (2) The images were registered to tissue probability maps and segmented into GM, WM and CSF; (3) The DARTEL method was used to nonlinearly normalize the segmented images to the Montreal Neurological Institute (MNI) spatial template; (4) In order to reduce the influence of spatial normalization, the nonlinear normalized image was modulated and the tissue volume was compensated. (5) The GM images were smoothed using a Gaussian kernel with a full width of 8 mm.

FSL (version 7.0.3) (https://fsl.fmrib.ox.ac.uk/fsl) was used for DTI image preprocessing. The specific steps were as follows:(1) Head motion correction and eddy-current correction; (2) Gradient direction correction; (3) Obtaining brain mask to remove non-brain tissue; (4) Tensor calculation: DTIFIT function was used to calculate the tensor, and the related scalar indexes of FA values were obtained. (5) Average FA images were generated, and then skeletonized images were used to create the skeleton of WM tracts, which were skeletonized using a FA threshold of 0.20.

In order to eliminate the scanner effects in different MRI equipments, we employed analysis of variance (ANOVA) to assess whether the MRI parameters of each sequence significantly differed among MRI scanners. If a parameter exhibited a significant difference, we used the ComBat method (https://github.com/Jfortin1/ComBatHarmonization) based on the Matlab platform (R2020b) to mitigate the heterogeneity. To evaluate the effect of harmonization, ANOVA was used to test the differences in MRI parameters among different scanners after harmonization. Details were provided in Supplementary material S2.

### 2.4 Statistical analysis

Data were analyzed using SPSS version 24.0 for Windows (SPSS Inc., Chicago, IL, USA). The normality of variable distributions was assessed with the Kolmogorov–Smirnov test. Variables that were normally distributed were presented as mean ± standard deviation, and overall group differences were evaluated using one-way ANOVA. Non-normally distributed variables were expressed as medians with interquartile ranges (IQR), and the Kruskal-Wallis test was employed for comparisons. Categorical variables were compared among groups using the chi-squared test. For multiple comparisons, Bonferroni correction was applied, and a two-tailed adjusted *p* < 0.05 was considered statistically significant.

Partial correlation analyses were performed to examine associations between MRI parameters [bilateral hippocampal (HIP) volume and hippocampal cingulum (CgH) FA] and AD pathology (CSF Aβ, P-tau) as well as cognitive composite scores (global cognition and memory), with age, sex, education, and APOE ε4 status included as covariates. The linear regression residual method was used to compute partial correlation coefficients. A total of 18 comparisons were tested, and the false discovery rate (FDR) procedure was applied to correct for multiple comparisons, with *P_FDR_* < 0.05 considered statistically significant. More details of the partial correlation were provided in Supplementary material S3.

VBM analysis was conducted using SPM12 software. The differences in gray matter volume (GMV) between groups 2–5 and group 1 were analyzed using the general linear model (GLM), with gender, age, and education level included as covariates. Statistical significance was determined using a cluster-defining threshold of *p* < 0.001 uncorrected at the voxel level, and clusters were considered significant at *p* < 0.05 after cluster-level family-wise error (FWE) correction for multiple comparisons (minimum cluster size = 200 voxels). The statistical results were visualized using the xjview plugin (https://www.alivelearn.net/xjview).

For the TBSS analysis, a voxel-wise statistical analysis of FA images was conducted using FSL software. The design matrix for two-sample t-tests comparing groups 2–5 with group 1 was constructed using the GLM tool, incorporating age, gender, and years of education as covariates. Statistical analyses were performed using permutation tests, with 5,000 permutations. Multiple comparisons correction was performed using Threshold-Free Cluster Enhancement (TFCE). The level of statistical significance was established at *p* < 0.05, corrected for FWE. Finally, the JHU ICBM-DTI-81 White-Matter Labels atlas was utilized for localization and quantitative analysis.

To further assess the robustness of primary findings, we performed a supplementary region of interest (ROI) analysis across 11 brain regions known to be affected in AD (Couvy-Duchesne et al., 2025; Chen Y. et al., 2023; Chen Q. et al., 2023). The GM ROIs comprised the bilateral HIP, amygdala (AMYG), and parahippocampal gyrus (PHG) from the AAL atlas. The WM ROIs comprised the splenium of corpus callosum (SCC), the genu of corpus callosum (GCC), and the body of corpus callosum (BCC), as well as the bilateral CgH, derived from the JHU ICBM-DTI-81 White-Matter Labels atlas. For each ROI, we performed a one-way ANCOVA with group (Group1-5) as the fixed factor and age, sex, and years of education as covariates (Model 1). Estimated marginal means and standard errors were extracted for each group, and the group effect size was quantified using partial eta-squared (η^2^). Given the significant differences in APOE ε4 carrier frequency across the five groups and its well-established influence on AD-related brain structural alterations, we performed sensitivity analyses to assess its potential confounding effect. Specifically, we constructed a second model (Model 2) by adding APOE ε4 status (carrier vs. non-carrier) as an additional covariate. The change in effect size was calculated as Δη^2^ = partial η^2^ (Model 2) - partial η^2^ (Model 1). To correct for multiple comparisons, we applied the FDR procedure across all 44 tests (11 regions of interest × 4 pairwise contrasts: Group 2–5 vs. Group 1). A *p-*value < 0.05 was considered statistically significant.

Additionally, to explore the APOE ε4 effect within each subgroup, we performed stratified ANCOVA separately for each group (Group 1–5), with APOE status as the independent variable while adjusting for age, sex, and years of education. The APOE ε4 effect was quantified as the standardized mean difference (Cohen’s d), derived from the regression coefficient divided by the root mean square error; 95% confidence intervals for Cohen’s d were computed using the model-based standard errors. To correct for multiple comparisons across all subgroup ROI combinations (5 groups × 11 ROIs = 55 tests), we applied the false discovery rate (FDR) procedure, with a significance threshold of *p* < 0.05.

## 3 Results

### 3.1 Demographics

A total of 178 subjects were enrolled, including 30 A-T- CN cases, 40 A + T- CN cases, 27 A + T + CN cases, 39 A + T + MCI cases, and 42 A + T + AD cases. There were significant differences in age, years of education, APOE ε4, MMSE, MoCA, WMS-LM, CDR, ADAS-Cog11, ADAS-Cog13, ADAS-Q4, RAVLT immediate, RAVLT learning, RAVLT forgetting, RAVLT percent forgetting among the five groups (*p* < 0.05), as shown in Table 1 and Supplementary Table S1.

**Table 1:** Comparison of demographic and neuropsychiatric scale data among five groups.

Variable	A-T- CN (G1)	A + T- CN (G2)	A + T + CN (G3)	A + T + MCI (G4)	A + T + AD (G5)	P value
*N*	30	40	27	39	42	
Age (years)	79.00 (72.75, 84.00)	74.00 (69.23, 77.75)	79.00 (75.00, 89.00)	75.00 (71.00, 78.00)	74.00 (66.00, 81.25)	0.004^c^
Gender, *N* (%)						0.915
Female	13 (43.33%)	18 (45.00%)	14 (51.85%)	16 (41.03%)	17 (40.48%)	
Male	17 (56.67%)	22 (55.00%)	13 (48.15%)	23 (58.97%)	25 (59.52%)	
Education (years)	18.00 (15.50, 19.00)	16.00 (15.00, 18.00)	16.00 (14.00, 18.00)	16.00 (13.00, 18.00)	15.50 (12.75, 16.00)	0.006^b^
APOE ԑ4, *N* (%)						<0.001^abde^
No	25 (83.33%)	25 (62.50%)	13 (48.15%)	7 (17.95%)	10 (23.81%)	
Yes	5 (16.67%)	15 (37.50%)	14 (51.85%)	32 (82.05%)	32 (76.19%)	
MMSE	29.00 (28.00, 30.00)	29.00 (28.00, 30.00)	29.00 (27.50, 30.00)	28.00 (26.00,29.00)	24.00 (22.75, 26.00)	<0.001^abdegh^
MoCA	24.50 (23.00, 27.25)	26.00 (24.00, 28.00)	24.00 (22.50, 26.50)	23.00 (21.00, 24.00)	19.00 (14.00, 20.25)	<0.001^bdegh^
WMS-LM	13.50 (10.75, 16.00)	13.00 (10.00, 15.75)	12.00 (11.00, 15.00)	6.00 (2.00, 9.00)	0.00 (0.00, 2.25)	<0.001^abdefg^
CDR	0.00 (0.00, 0.00)	0.00 (0.00, 0.00)	0.00 (0.00, 0.00)	1.00 (0.50, 2.00)	4.50 (3.50, 6.00)	<0.001^abdefgh^
ADAS-Cog11	3.00 (3.00, 6.75)	4.50 (3.33, 7.00)	6.00 (3.00, 8.50)	11.00 (8.00, 14.00)	18.00 (14.00, 23.50)	<0.001^abdefgh^
ADAS-Cog13	7.00 (4.83, 10.17)	8.00 (5.00, 10.00)	10.00 (7.00, 14.00)	18.00 (13.00, 24.00)	27.00 (23.00,36.00)	<0.001^abdefgh^
ADAS-Q4	2.00 (1.00, 3.00)	3.00 (2.00, 4.00)	3.00 (2.00, 5.00)	6.00 (4.00, 9.00)	9.00 (7.75, 10.00)	<0.001^abdefg^
RAVLT immediate	50.00 (41.75, 57.00)	47.00 (39.25, 53.00)	39.00 (31.50, 49.50)	30.00 (26.00, 36.00)	23.50 (17.75, 28.50)	<0.001^abdefg^
RAVLT learning	6.00 (5.00, 8.25)	6.50 (5.00, 7.00)	6.00 (3.00, 7.00)	3.00 (2.00, 5.00)	2.00 (0.75, 3.00)	<0.001^abdegh^
RAVLT forgetting	3.00 (1.00, 5.00)	3.00 (1.00, 6.00)	4.00 (2.00, 5.00)	5.00 (3.00, 6.00)	4.00 (3.00, 6.00)	0.025^a^
RAVLT percent forgetting	25.00 (11.65, 38.46)	27.27 (7.28, 49.17)	44.44 (14.84, 59.03)	75.00 (40.00, 100.00)	100.00 (77.08, 100.00)	<0.001^abdeg^

G, group; MMSE, mini mental state examination; MOCA, montreal cognitive assessment; WMS-LM, Wechsler Memory Scale logical memory; ADAS, Alzheimer’s Disease Assessment Scale; RAVLT, rey auditory-verbal learning test.

The chi-square test was used for the nominal variable. The Kruskal-Wallis test was used for the continuous variable with non-normal distribution. ^a^ Statistically significant difference between Group 1 versus Group 4, ^b^ Statistically significant difference between Group 1 versus Group 5, ^c^ Statistically significant difference between Group 2 versus Group 3, ^d^ Statistically significant difference between Group 2 versus Group 4, ^e^ Statistically significant difference between Group 2 versus Group 5, ^f^ Statistically significant difference between Group 3 versus Group 4, ^g^ Statistically significant difference between Group 3 versus Group 5, ^h^ Statistically significant difference between Group 4 versus Group 5.

### 3.2 VBM analysis results

**Figure 1:**
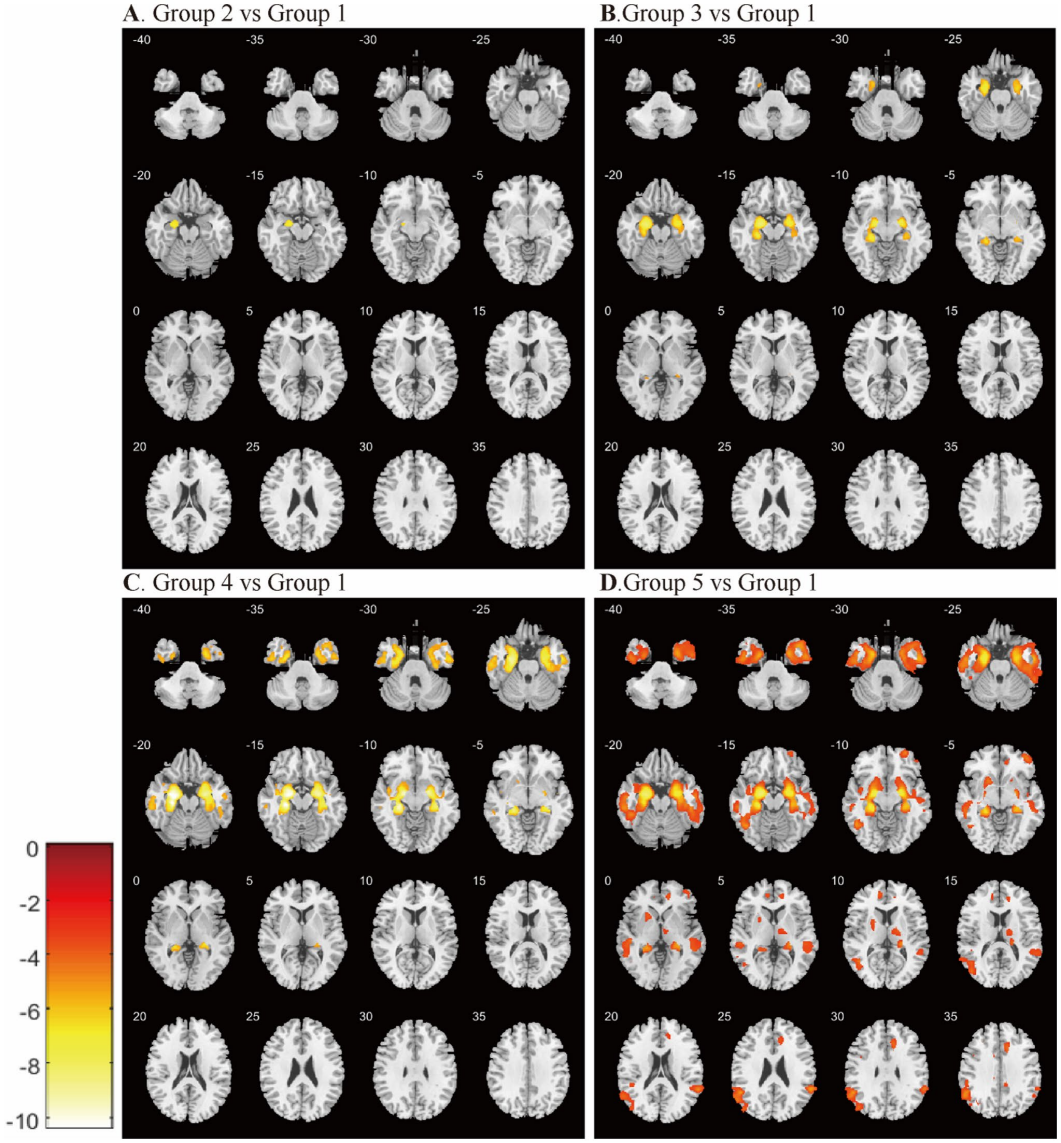
VBM analysis of gray matter volume (GMV) differences between groups 2−5 and group 1. The figure consists of four panels **(A−D)**, corresponding to groups 2, 3, 4, and 5 compared with group 1, respectively (i.e., **A**: group 2 vs. 1; **B**: group 3 vs. 1; **C**: group 4 vs. 1; **D**: group 5 vs. 1). GMV differences were assessed using a general linear model (GLM) in SPM12, with age, sex, and education level entered as covariates. A cluster defining threshold of *P* < 0.001 (uncorrected) was applied at the voxel level, and clusters were considered significant after cluster-level family-wise error (FWE) correction at *P* < 0.05, with a minimum cluster size of 200 voxels. Hot colors indicate regions where GMV was significantly decreased in groups 2−5 relative to group 1. The color bar represents *t* statistic values.

Compared with Group 1, Groups 2–5 showed varying degrees of GM atrophy (Table 2 and Figure 1). Compared with Group 1, Group 2 exhibited initial GM atrophy in the left HIP. In Group 3, GM atrophy extended to bilateral HIP, bilateral parahippocampal gyrus (PHG), bilateral amygdala (AMYG), and the left fusiform gyrus (FFG). Group 4 demonstrated further GM atrophy in the right fusiform gyrus (FFG), bilateral temporal pole: superior temporal gyrus (TPOsup), right temporal pole: middle temporal gyrus (TPOmid), right middle temporal gyrus (MTG), right inferior temporal gyrus (ITG), and right lenticular nucleus-pallidum (PAL). Group 5 showed additional gray matter atrophy in the bilateral inferior parietal gyrus (IPG), left angular gyrus (ANG), bilateral lenticular nucleus-putamen (PUT), bilateral supramarginal gyrus (SMG), bilateral superior temporal gyrus (STG), left middle occipital gyrus (MOG), right postcentral gyrus (PoCG), bilateral middle frontal gyrus (MFG), bilateral median cingulate and paracingulate gyrus (MCC), right thalamus (THA), left medial superior frontal gyrus (SFGmed), left dorsolateral superior frontal gyrus (SFGdor), and right precuneus (PCUN).

**Table 2:** Brain regions with differences in GMV between groups 2–5 compared to group 1.

Group	Cluster	Voxels	Brain Region	MNI			*t* value	PFWE
				X	Y	Z		
G2 vs. G1	1	399	L: HIP	−24	−7	−18	−6.6781	0.020
G3 vs. G1	1	3,073	L: HIP/PHG/AMYG/FFG	−27	−15	−17	−8.1383	<0.001
	2	1978	R: HIP/PHG/AMYG	26	−11	−16	−7.3991	<0.001
G4 vs. G1	1	7,562	R: HIP/PHG/FFG/TPOmid/TPOsup	32	−10	−23	−8.4569	<0.001
	2	6,531	L: HIP/FFG/PHG/AMYG/TPOsup	−28	−13	−19	−9.1221	<0.001
	3	1,442	L: MTG/ITG	−57	−11	−24	−6.0968	<0.001
	4	1,367	R: MTG/ITG/PAL	56	−15	−27	−4.9798	<0.001
G5 vs. G1	1	21,778	L: HIP/ITG/MTG/FFG/IPG/PHG/ANG/PUT/TPOsup/SMG/AMYG/STG/MOG	−23	−9	−17	−8.1383	<0.001
	2	19,171	R: HIP/ITG/PHG/FFG/MTG/TPOmid/STG/SMG/TPOsup/AMYG/IPG/PoCG/PUT	40	−16	−15	−7.3991	<0.001
	3	841	R: MFG	27	57	−12	−4.5113	<0.001
	4	1,023	B: MCC	11	21	33	−4.0800	<0.001
	5	621	R: THA	18	−10.5	12	−3.9145	0.017
	6	260	L: SFGmed	−12	49.5	9	−3.8084	0.025
	7	699	L: MFG/SFGdor	−33	19.5	43.5	−4.5867	0.013
	8	238	R: PCUN	10.5	−46.5	57	−3.9440	0.033

G, group; L, left; R, right; B, bilateral; HIP, Hippocampus; PHG, parahippocampal gyrus; AMYG, amygdala; FFG, fusiform gyrus; TPOmid, temporal pole: middle temporal gyrus; TPOsup, temporal pole: superior temporal gyrus; MTG, middle temporal gyrus; ITG, inferior temporal gyrus; PAL, lenticular nucleus-pallidum; IPG, inferior parietal, but supramarginal and angular gyrus; ANG, angular gyrus; PUT, lenticular nucleus-putamen; SMG, supramarginal gyrus; STG, superior temporal gyrus; MOG, middle occipital gyrus; PoCG, postcentral gyrus; MFG, Middle frontal gyrus; MCC, median cingulate and paracingulate gyrus; THA, thalamus; SFGmed, superior frontal gyrus, medial; SFGdor, superior frontal gyrus, dorsolateral; PCUN, precuneus.

### 3.3 TBSS analysis results

**Figure 2:**
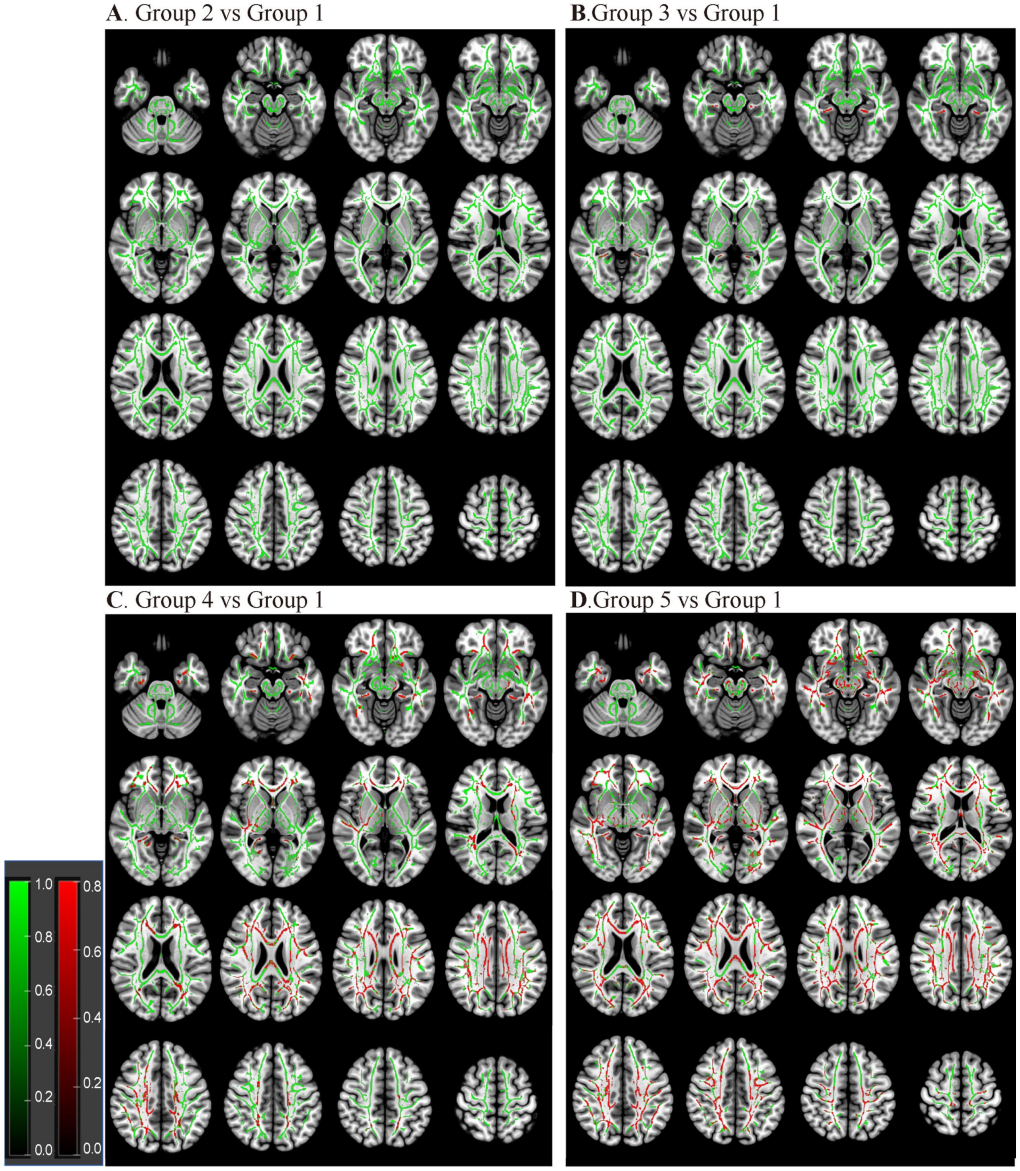
TBSS analysis of fractional anisotropy (FA) differences between groups 2−5 and group 1. The figure consists of four panels **(A−D)**, corresponding to groups 2, 3, 4, and 5 compared with group 1, respectively (i.e., **A**: group 2 vs. 1; **B**: group 3 vs. 1; **C**: group 4 vs. 1; **D**: group 5 vs. 1). Voxel-wise comparisons were performed in FSL using a general linear model (GLM) with age, sex, and years of education as covariates. Statistical significance was determined by permutation testing (5000 permutations) with threshold-free cluster enhancement (TFCE), and the resulting maps were corrected for family-wise error (FWE) at *P* < 0.05. Green represents the mean FA skeleton of all participants. Red indicates regions with significantly decreased FA in groups 2−5 relative to group 1. The color bar represents the corresponding range of FA values.

Compared with Group 1, Groups 2–5 showed varying extent of WM fiber tracts alterations (Table 3 and Figure 2). Compared with Group 1, Group 2 exhibited no significant reduction in FA values. The decrease in FA values was observed exclusively in Group 3, predominantly located in the bilateral CgH. Group 4 exhibited a significant reduction in FA values. The primary affected WM fiber tracts included the bilateral superior longitudinal fasciculus (SLF), bilateral sagittal stratum (SS), left uncinate fasciculus (UF), bilateral posterior thalamic radiation (PTR), bilateral superior corona radiata (SCR), bilateral posterior corona radiata (PCR), anterior corona radiata (ACR), right external capsule (EC), left anterior limb of internal capsule (ALIC), bilateral posterior limb of internal capsule (PLIC), right retrolenticular part of the internal capsule (RLIC), left cerebral peduncle (CP), fornix (FX), SCC, GCC, and BCC. In Group 5, both the number of voxels and the extent of differential WM fiber tracts were further increased, with additional involvement of the right UF, left EC, right ALIC, left RLIC and right CP.

**Table 3:** WM fiber tracts with differential FA values in groups 2–5 compared with group 1.

Group	Cluster	Voxels	Tract	MNI			PFWE
				X	Y	Z	
G2 vs. G1	None						
G3 vs. G1	1	1,181	CgH. B	5	−22	−15	0.040
G4 vs. G1	1	14,092	CgH. B/GCC/BCC/ SCC/SLF. B/SS. B/UF. L/PTR. B/PCR. B/SCR. B/ACR. B/EC. R/ALIC. L/PLIC. B/RLIC. R/FX/CP. L	2	−6	30	0.020
G5 vs. G1	1	23,607	CgH. B/GCC/BCC/SCC/SLF. B/SS. B/UF. B/PTR. B/PCR. B/SCR. B/ACR. B/EC. B/ALIC. B/PLIC. B/RLIC. B/FX/CP. B	19	23	24	<0.001

G, group; B, bilateral; L, left; R, right; CgH, hippocampal cingulum; SLF, superior longitudinal fasciculus; SS, sagittal stratum (include inferior longitudinal fasciculus and inferior fronto-occipital fasciculus); UF, uncinate fasciculus; SCC, splenium of corpus callosum; PTR, posterior thalamic radiation; PCR, posterior corona radiata; SCR, superior corona radiata; GCC, genu of corpus callosum; BCC, body of corpus callosum; ACR, anterior corona radiata; EC, external capsule; ALIC, anterior limb of internal capsule; PLIC, posterior limb of internal capsule; RLIC, retrolenticular part of the internal capsule; FX, fornix; CP, cerebral peduncle.

### 3.4 Results of correlation analysis

**Figure 3:**
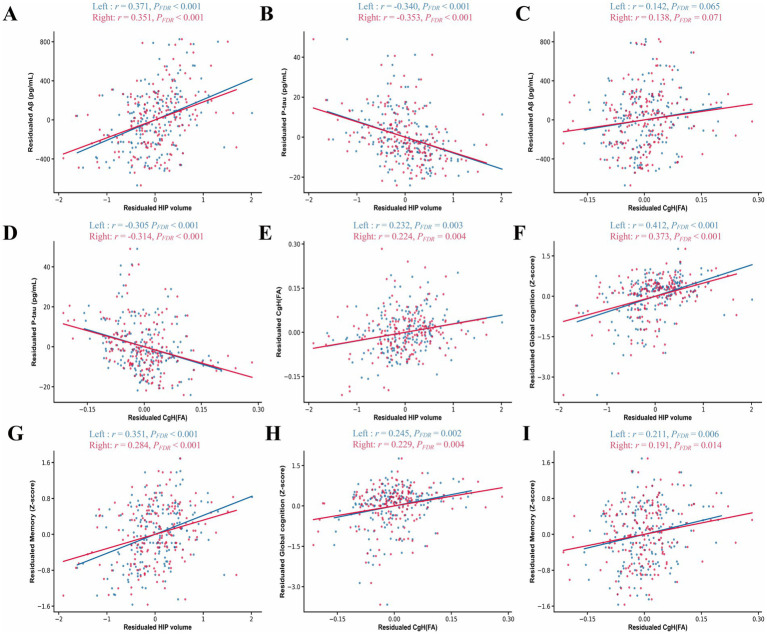
Partial correlation scatter plots between MRI parameters, AD pathology, and cognitive function (residualized data). The figure consists of nine panels **(A−I)**, with the following variable pairs plotted in each panel: **(A)** CSF Aβ level vs. HIP volume; **(B)** CSF P-tau level vs. HIP volume; **(C)** CSF Aβ level vs. CgH FA value; **(D)** CSF P-tau level vs. CgH FA value; **(E)** CgH FA value vs. HIP volume; **(F)** Global cognition vs. HIP volume; **(G)** Memory vs. HIP volume; **(H)** Global cognition vs. CgH FA value; and **(I)** Memory vs. CgH FA value. All variables were residualized for age, sex, years of education, and APOE ε4 status. Blue dots and regression lines represent the left hemisphere; red denotes the right hemisphere. Regression slopes and intercepts were calculated from the residualized data. For each panel, partial correlation coefficient (*r*), 95% confidence interval (CI), uncorrected *P* values, and false discovery rate (FDR) adjusted *P* values are reported in Supplementary Table S2.

Partial correlation analyses (Figure 3 and Supplementary Tables S2) showed that bilateral HIP volume correlated with CSF Aβ (left: *r* = 0.371; right: *r* = 0.351, both *P_FDR_* < 0.001) and P-tau (left: *r* = −0.340; right: *r* = −0.353, both *P_FDR_* < 0.001). CGH FA value correlated with P-tau (left: *r* = −0.305; right: *r* = −0.314, both *P_FDR_* < 0.001) but not with Aβ (*P_FDR_* > 0.05). HIP volume correlated with ipsilateral CGH FA value (left: *r* = 0.232, *P_FDR_* = 0.002; right: *r* = 0.224, *P_FDR_* = 0.004). HIP volume correlated with global cognition (left: *r* = 0.412; right: *r* = 0.373, both *P_FDR_* < 0.001) and memory (left: *r* = 0.351; right: *r* = 0.284, both *P_FDR_* < 0.001). CGH FA value correlated with global cognition (left: *r* = 0.245, *P_FDR_* = 0.002; right: *r* = 0.229, *P_FDR_* = 0.004) and memory (left: *r* = 0.211, *P_FDR_* = 0.006; right: *r* = 0.191, *P_FDR_* = 0.014).

### 3.5 ROI analysis and APOE ε4 sensitivity analysis

**Figure 4:**
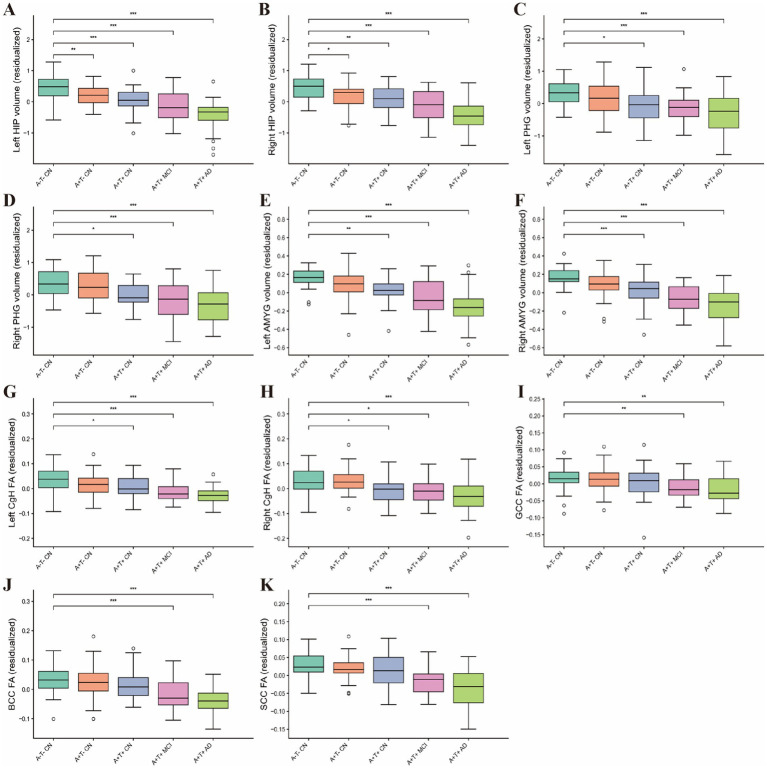
Residualized ROI distributions across 5 groups for Model 1 (without APOE adjustment). The figure consists of eleven panels **(A−K)**, with panels **(A,B)** showing left and right HIP volumes, respectively; **(C,D)** showing left and right PHG volumes, respectively; **(E,F)** showing left and right AMYG volumes, respectively; **(G,H)** showing left and right CgH FA values, respectively; and panels **(I−K)** showing GCC, BCC, and SCC FA values, respectively. Boxplots display residualized values after adjusting for age, sex, and years of education. Significance vs. G1: *P < 0.05, **P < 0.01, ***P < 0.001 (FDR-corrected). Full ANCOVA statistics are reported in Supplementary Table S3.

**Figure 5:**
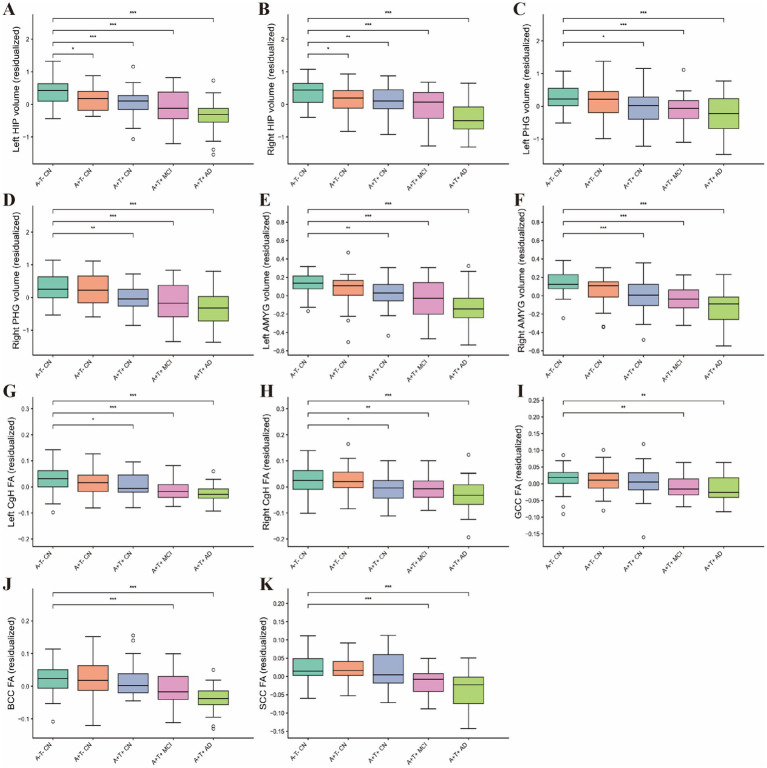
Residualized ROI distributions across 5 groups for Model 2 (with APOE adjustment). The figure consists of eleven panels **(A−K)**, with panels **(A,B)** showing left and right HIP volumes, respectively; **(C,D)** showing left and right PHG volumes, respectively; **(E,F)** showing left and right AMYG volumes, respectively; **(G,H)** showing left and right CgH FA values, respectively; and panels **(I−K)** showing GCC, BCC, and SCC FA values, respectively. Boxplots display residualized values after adjusting for age, sex, years of education, and APOE ε4 status. Significance vs. G1: *P < 0.05, **P < 0.01, ***P < 0.001 (FDR-corrected). Full ANCOVA statistics and Δη2 are reported in Supplementary Table S4.

The ANCOVA results for the 11 ROIs are summarized in Supplementary Tables S3 (Model 1) and S4 (Model 2). In Model 1, all 11 ROIs showed significant group differences across the five groups (all *p* < 0.001), with partial η^2^ ranging from 0.116 to 0.409. Adding APOE ε4 status as a covariate led to a slight reduction in the partial η^2^ for the AT(N) group effect across all 11 ROIs, with Δη^2^ ranging from −0.033 to −0.001 (Supplementary Table S4). Post-hoc pairwise comparisons in Model 1 (Figure 4) and Model 2 (Figure 5) against Group 1 revealed a region-specific progressive pattern across all 11 ROIs (all *P_FDR_* < 0.05). Bilateral HIP volumes showed significant reductions in Group 2–5. Bilateral PHG volumes, AMYG volumes, and CgH FA values showed significant differences in Group 3–5. Corpus callosum (GCC, BCC, SCC) FA values showed significant reductions in Group 4 and Group 5.

**Figure 6:**
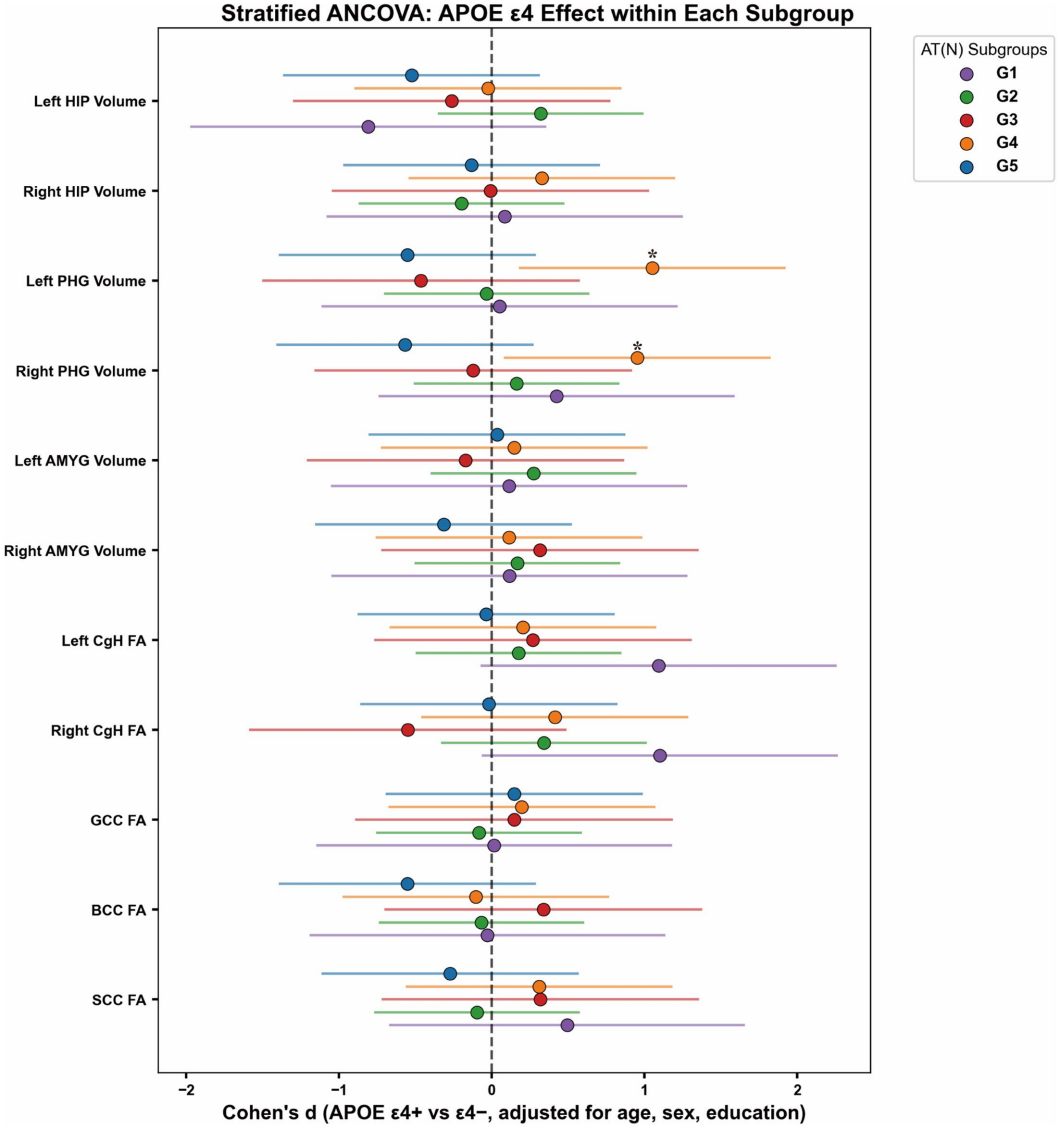
Forest plot of APOE ε4 effects within each subgroup across 11 ROIs. Forest plot displaying effect sizes (Cohen’s *d*) and 95% confidence intervals for APOE ε4 carrier status (carriers vs. non-carriers) within each subgroup, derived from ANCOVA models adjusted for age, sex, and education. Each point represents the Cohen’s *d* for a specific ROI within a given subgroup; error bars indicate 95% confidence intervals. The vertical dashed line at *X* = 0 indicates the null effect (no difference between APOE ε4 carriers and non-carriers). All comparisons were non-significant after FDR correction for 55 tests (*PFD_R_* > 0.05 for all). * indicates nominal uncorrected *p* < 0.05 (not significant after FDR correction). Full statistical details (*F* statistics, *p* values, *PFD_R_* values) are provided in Supplementary Table S5.

In the stratified within-subgroup analysis, no significant independent APOE effect was detected after FDR correction. Two nominal uncorrected differences were noted in the Group 4 [left PHG: Cohen’s d = 1.051, 95% CI (0.178, 1.924), uncorrected *p* = 0.020; right PHG: Cohen’s d = 0.952, 95% CI (0.079, 1.826), uncorrected *p* = 0.034]. However, after FDR correction, all adjusted *p* values remained non-significant (Supplementary Table S5 and Figure 6).

## 4 Discussion

In this study, we constructed a cross-sectional cohort of AD based on the AT(N) pathological staging model to investigate the relationship between AD pathological changes and brain structural alterations. Our findings revealed that GM atrophy initially emerged in the A + T- stage, specifically in the HIP. In contrast, WM fiber tract alterations were first observed in the A + T + stage, with lesions situated in the CgH adjacent to the HIP. In the MCI and AD groups, structural abnormalities were detected across broader GM and WM regions. The observed spatial distribution of brain changes across stages is consistent with the P-tau transmission hypothesis from an in vivo perspective. Importantly, the N biomarker was excluded from the grouping criteria and served as the primary outcome, while group assignment was based exclusively on A/T biomarkers and independent of N status. The observed spatial progression of GM atrophy and WM alterations across the A-T-, A + T-, and A + T + stages indicates that the presence of both Aβ and tau pathology is associated with increasingly extensive structural alterations, elucidating the relationship between core AD pathology and N-type changes.

VBM analysis revealed that in the early stage of AD (A + T- CN), GM atrophy was predominantly localized to the HIP. The HIP is recognized as the initial site of AD pathology, where Aβ deposition is thought to initiate a cascade of neuronal pathological changes (Xhima et al., 2024; Perosa et al., 2024). Similarly, our results indicated that HIP volume was associated with Aβ levels. In addition, HIP volume correlated with global cognitive function and memory performance, which may suggest that HIP atrophy contributes to cognitive decline. However, some studies (Li et al., 2020; Tardif et al., 2018) suggest that HIP volume may paradoxically increase in the early stages of AD. This phenomenon could be attributed to compensatory mechanisms such as neuronal proliferation and synaptic remodeling, which temporarily counteract the effects of Aβ accumulation, thereby maintaining or even increasing HIP volume (Leng and Edison, 2021). By contrast, HIP volume results in this study are consistent with the findings of Pan et al. (2025). The inconsistent HIP volume changes across cohorts likely arise from differing stages of compensatory and decompensatory Aβ pathology. Previous longitudinal studies (Pegueroles et al., 2017; Montal et al., 2021) found the GM structure exhibited early compensatory enlargement that shifted to progressive atrophy with advancing Aβ pathology. We attribute this discrepancy primarily to differences in cohort characteristics. Our A + T- CN sample, compared to cohorts reporting HIP volume enlargement, may have a relatively older age and higher Aβ burden, all of which can accelerate the exhaustion of compensatory reserve. Consequently, the A + T- subjects in this study may have already transitioned out of compensatory remodeling into early neurodegeneration. Previous studies (Chiu et al., 2025; Rajesh and Kanneganti, 2022) have also suggested sustained Aβ accumulation is associated with neuronal dysfunction and downstream neurodegenerative changes.

In A + T + CN subjects, GM atrophy extended to structures adjacent to the HIP, encompassing the PHG, AMYG, and FFG. Elevated P-tau burden has been previously documented to coincide with GM atrophy in AD cohorts (Wang et al., 2023). Accordingly, we speculate that abnormal P-tau accumulation may correlate with the regional GM atrophy observed in A + T + CN subjects. Among these structures, the PHG, situated on the lateral side of the HIP, plays a crucial role in memory and spatial cognition by participating in the encoding and retrieval of environmental information and memories (Lin et al., 2021). The PHG shares close anatomical and functional connections with the HIP, and their degeneration in AD typically occurs concurrently (Valdés Hernández et al., 2020). The AMYG is implicated in emotion regulation, memory formation, and social behavior, maintaining extensive neural connections with the HIP, prefrontal cortex, and other regions, which are essential for emotional and mnemonic functions (Meisner et al., 2022). Positioned between the ITG and the PHG, the FFG is primarily involved in higher-order cognitive processes such as face recognition, visual processing, and olfactory perception (Chen et al., 2021; Ma et al., 2020). Previous studies (Sala et al., 2020; Aggleton and O'Mara, 2022) have also indicated that brain regions closely associated with the HIP exhibit early atrophy, which correlates strongly with patients’ cognitive decline. The observed GM atrophy spatial pattern is consistent with the pathological cascade hypothesis, which posits that tau pathology propagates outward from the HIP to its anatomically adjacent brain regions.

In A + T + MCI subjects, GMV atrophy was not limited to the medial temporal lobe but also extended to the PAL and throughout the temporal lobe, encompassing regions such as TPOmid, TPOsup, MTG, and ITG. For A + T + AD subjects, GM atrophy extended further, affecting not only the aforementioned areas but also expanding to the THA, parietal lobe, frontal lobe, occipital lobe, and MCC. Previous research has demonstrated that thalamic damage associated with AD primarily affects the anterior thalamic nuclei (Zhao et al., 2024). These nuclei are interconnected with the HIP, temporal lobe, and frontal lobe through the FX. This network establishes the foundation of a tripartite memory system comprising the anterior thalamic nuclei, HIP, and cortex, wherein the integrity of these connections is essential for optimal episodic memory function. Moreover, GM atrophy in the parietal, frontal, and occipital lobes is linked to impairments in language, spatial perception, memory, and executive function, with reductions in these regions signifying a more advanced stage of the disease (Zhang et al., 2024; Pini et al., 2016). The observed GM atrophy pattern in this study aligns with previous findings (Haller et al., 2023; Racine et al., 2014) in which HIP atrophy occurred in early AD, while temporal, parietal, frontal, and global GM atrophy was seen in advanced stages.

The underlying neural mechanism of WM fiber tracts alterations was hypothesized to involve demyelination and neuronal loss, with FA serving as a marker of structural integrity (Navarro et al., 2018). In this study, we observed no significant change in the FA values of WM tracts among A + T- CN subjects. This observation may align with the view that, while Aβ protein accumulation can induce a series of neurotoxic effects, tau protein has not yet accumulated sufficiently to cause extensive neuronal damage, thereby maintaining relatively intact WM tracts (Chen Q. et al., 2023; Terao et al., 2025). Our results revealed that CgH FA values were associated with tau levels, which tentatively suggests a closer association between tau pathology and WM integrity, consistent with the above interpretation. Nevertheless, given the modest effect size (*r* = −0.305; −0.314), this finding should be interpreted with caution. Moreover, FA lacks biological specificity and may reflect multiple pathological processes. Future longitudinal studies incorporating additional diffusion metrics, such as mean diffusivity (MD), radial diffusivity (RD), and axial diffusivity (AD), may offer a novel perspective on the sequence and nature of these microstructural changes.

However, in A + T + CN subjects, the CgH was the only WM tract to exhibit reduced integrity. In addition, correlation analysis revealed modest correlations between CgH FA value and HIP volume as well as P-tau levels, consistent with potential interrelations between tau pathology and brain structural alterations. This spatial pattern is consistent with the hypothesis that tau pathology may spread from the HIP to adjacent WM tracts. Nevertheless, cross-sectional data cannot validate directional pathological propagation, and longitudinal follow-up studies are required to verify this inference. Chen Q. et al. (2023) similarly reported a significant reduction in FA values of the CgH in A + T + CN subjects compared to A + T- CN subjects. Furthermore, their study found a consistent and sustained decrease in FA values in both MCI and AD patients, aligning with our findings. Several studies (Jacobs et al., 2018; Lee et al., 2015) have shown that tau levels in AD pathology correlate with CgH FA values, predict downstream tau deposition in Aβ + subjects, and are associated with cognition. Additionally, we also observed that FA values in the CgH were correlated with global cognition and memory. Collectively, these cross-sectional correlations indicate close links between CgH microstructure, tau burden and cognition along the AD continuum, while longitudinal data are needed to clarify directional effects. And in A + T + CN subjects, CgH tract alterations and GMV atrophy extended to other regions of the temporal lobe. These findings, consistent with prior research (Delvenne and Malloy, 2025), indicate that P-tau may serve as a critical pathological factor in the progression of AD.

In the A + T + MCI and AD groups, extensive WM alterations were observed in the UF, GCC, BCC, SCC, PTR, PCR, SLF, SCR, ACR, EC, ALIC, PLIC, RLIC, FX, and CP. The corpus callosum (CC), as the largest commissural fiber tract in the brain, coordinates the transmission of sensory, motor, cognitive, and emotional information between the two cerebral hemispheres (Sang et al., 2022). The SLF is the largest association fiber that connects different lobes within the ipsilateral cerebral hemisphere and is associated with the transmission of multiple types of sensory information (Di Carlo et al., 2019). Consequently, in AD patients, impairment of CC and SLF significantly impairs information transmission between and within hemispheres. In addition to SLF, the SS and UF are also important association fibers. The SS originates posterior to the inferotemporal sulcus of the insula and terminates in the posterior temporal-occipital-parietal cortex, primarily comprising the inferior longitudinal fasciculus (ILF) and the inferior fronto-occipital fasciculus (IFOF) (Lin et al., 2020). The UF connects the medial temporal lobe structures (inferior, middle, and superior temporal gyrus) with the frontal lobe (Zhang et al., 2018). ILF, IFOF, and UF together form the ventral stream, which plays a crucial role in semantic processing functions such as language comprehension, naming, and word visual processing (Park et al., 2019). Previous research (Zhong et al., 2023; Gao et al., 2024) has shown that cognitive impairment in patients correlates with impaired SS and UF integrity, affecting memory and other cognitive functions. The FX is a critical projection fiber connecting the HIP formation to basal forebrain structures and mammillary bodies, and its degeneration is closely associated with episodic memory impairment in AD (Madhavan et al., 2016). The EC and internal capsule (IC) are projection fibers; the EC connects the claustrum to neighboring cortical areas, while the ALIC primarily carries fronto-thalamic projections. In contrast, PLIC, and RLIC serve as major conduits for motor, sensory, and visual pathways, respectively. And their integrity decline correlates with cognitive deterioration in AD and MCI patients (Li et al., 2025; Guo et al., 2026). PTR, PCR, SCR, ACR, and CP all belong to projection fibers, which are major nerve fiber tracts connecting the cortex and subcortical regions, responsible for long-distance information transmission, advanced cognitive function, and motor control (Menegaux et al., 2017; Burke et al., 2023; Chan et al., 2019). In AD and MCI patients, reduced integrity of these fibers leads to various functional disorders and cognitive impairments (Biesbroek et al., 2024).

In summary, the CgH was specifically affected in the A + T + CN stage. In the A + T + MCI and A + T + AD stages, more extensive WM fiber alterations occurred, impairing the function of these fiber tracts in transmitting information between different brain regions, thereby exacerbating cognitive, executive, and motor control deficits. Therefore, pathological damage in AD is not limited to specific GM regions but broadly affects the brain’s white matter network, particularly key nerve fiber pathways, leading to a wide range of clinical manifestations. Our observations of WM microstructural alterations align with emerging evidence on perivascular pathway involvement in AD. Yu et al. (2025) recently reported that the DTI-ALPS index, a valid proxy metric for glymphatic function, decreases significantly in patients with AD relative to HC/MCI (*p* < 0.001) and correlates strongly with CSF Aβ_42_ (*r* = 0.78) and inversely with P-tau_181_ (*r* = −0.683, *p* < 0.001). These findings indicate that impaired perivascular clearance may synergistically exacerbate the WM degeneration patterns identified in our cohort, particularly during advanced disease stages. Collectively, these results support a mechanistic model whereby vascular dysfunction and tau pathology cooperatively drive structural deterioration.

ROI analyses across 11 ROIs were generally consistent with the spatial patterns observed in VBM and TBSS, with HIP volume alterations in the A + T- CN group, CgH FA value alterations in A + T + CN group, and widespread GM and WM involvement in MCI an AD stages (partial η^2^ = 0.116–0.409). After adjusting for APOE ε4, group effect sizes decreased minimally (Δη^2^ = −0.033 to −0.001). Stratified analyses did not detect significant independent APOE ε4 effects after FDR correction, though nominal uncorrected differences were noted in PHG (G4). These findings suggest that APOE ε4 may have only a limited confounding role in our cohort, and that the structural progression observed may be more closely linked to A/T status than to genetic background. However, structural degeneration across the AD spectrum may be mediated by multiple factors. Cerebrovascular pathologies such as cerebral small vessel disease often coexist with AD in the elderly population and can independently induce structural changes (Biesbroek et al., 2024). In addition, age-related neurodegenerative changes may synergize with AD pathology to exacerbate brain tissue changes (Pini et al., 2016). Therefore, caution is warranted in interpreting the present findings, and further validation through future studies is needed.

Despite this study’s initial insights into the changes in brain structures across the AD continuum, several limitations remain. First, this study adopted a cross-sectional design. Although we stratified participants by A/T biomarker status to approximate disease progression, cross-sectional data cannot establish causal relationships between AD pathology and structural changes or definitively determine the directional spread of AD neuropathology over time. Longitudinal follow-up data will be required to verify such sequential pathological trajectories. Second, examining N biomarkers such as GM atrophy and WM alterations as outcomes in subjects grouped by A and T biomarkers may introduce potential circularity. The observed structural differences may partially reflect the N component itself. Thus, our findings should be interpreted as associations between A/T pathology and N-type changes, rather than independent validation of the AT(N) framework. Future longitudinal studies with repeated follow-up MRI and biomarker assessments could effectively disentangle these complex relationships by clarifying the temporal sequence of pathological changes. Such designs would enable us to determine the directional predictive effects of baseline Aβ and tau pathology on subsequent progressive N-type structural degeneration, thereby eliminating the circular bias inherent in cross-sectional observations. Third, in TBSS analysis, the spatial smoothing of WM skeletons and the setting of statistical thresholds may cause anatomically distinct areas of lesion to merge into a single cluster due to spatial continuity. To minimize the loss of information, the entire fiber bundles included in the cluster were analyzed. As our study focused on global WM alterations rather than hemispheric asymmetries, the TBSS whole-brain analysis was performed without separating left and right hemispheres. This approach, however, may mask subtle lateralized changes. In addition, FA lacks biological specificity and may reflect a range of pathological processes. The absence of MD, RD, and AD in the current dataset limits our ability to fully characterize the microstructural abnormalities. Finally, although our sensitivity analyses suggested that APOE ε4 status did not substantially confound the observed group differences, the possibility of residual or interactive effects cannot be fully excluded in the current cohort, and larger studies are warranted to further validate these findings.

## 5 Conclusion

In this study, we characterized the spatial patterns of GM atrophy and WM microstructural alterations across biologically defined stages of the AD continuum using VBM and TBSS. In the A + T- stage, GM atrophy was predominantly observed in the HIP. In the A + T + stage, GM atrophy extended to adjacent medial temporal structures, whereas WM integrity reduction was detectable in the CgH. In the A + T + MCI and A + T + AD stages, structural changes involved broader GM regions and widespread WM tracts. These cross-stage spatial patterns are consistent with the hypothesized role of tau pathology in the topographic progression of AD-related structural alterations. However, given the cross-sectional design, causal relationships or definitive propagation directions cannot be established. Our findings highlight the spatial association between A/T pathological states and N-type structural outcomes, providing imaging evidence that may inform early identification and stratification of AD-related neurodegeneration.

## Data Availability

The original contributions presented in the study are included in the article/Supplementary material, further inquiries can be directed to the corresponding author/s.
