# Supplementary material for: Structural changes in gray matter and white matter across the Alzheimer’s disease continuum
Source: Front Neurosci. 2026 Aug 5;20:1853132. doi: 10.3389/fnins.2026.1853132 (PMC13487739; doi:10.3389/fnins.2026.1853132)
Supplement: Supplementary file 1 [file Table_1.DOCX]

**Title:** Structural Changes in Gray Matter and White Matter across the Alzheimer‘s Disease Continuum

**Manuscript ID:** 1853132

**Supplementary Materials**

**Supplementary Methods S1.** Calculation of cognitive composite scores

The Global cognition composite was derived from the MMSE, MoCA, and ADAS-Cog13 to capture overall cognitive function across multiple domains. The Memory composite was derived from WMS-LM, RAVLT immediate, RAVLT learning, and RAVLT percent forgetting to index long-term, short-term, immediate memory, learning memory and retention capacity, respectively. Global cognition and Memory composite scores were calculated as follows. First, raw scores of MMSE, MoCA, and ADAS-Cog13 (reverse-coded so that higher scores indicate better performance) were converted to Z-scores using the mean and standard deviation of the cognitively normal (CN) subgroup at baseline. The Global cognition score was then computed as the average of these three Z-scores. Similarly, the Memory score was calculated as the average of the Z-scores of WMS-LM, RAVLT immediate, RAVLT learning, and RAVLT percent forgetting (reverse-coded so that lower scores indicate better performance) using the same CN-based standardization. All composite scores were subsequently re-standardized to have a mean of 0 and standard deviation of 1 across the entire sample for interpretability.

**Supplementary Methods S2.** ComBat harmonization

In the ComBat harmonization procedure, we applied voxel-wise corrections to FA and GMV maps that had been spatially normalized to standard space (FMRIB58_FA standard space and MNI152 space). For each participant, the normalized image (in NIFTI format) and the corresponding masks were input into the model, with scanner designated as the batch variable and age, sex, education years, and group as covariates to be preserved. The harmonized data were then reconstructed into 3D NIFTI files for each subject. To evaluate effectiveness, we extracted FA values from WM tracts using the JHU ICBM-DTI-81 White-Matter Labels atlas, and GMV values from brain regions using the AAL atlas. For each ROI, we applied a one-way ANOVA with scanner as the independent variable, and calculated the partial eta-squared (η^2^) for the scanner factor to quantify the proportion of variance explained by scanner differences. All ROI-wise ANOVA results were corrected for multiple comparisons using the false discovery rate (FDR) at *P* < 0.05. Before harmonization, significant scanner-related differences were observed across multiple ROIs (partial η^2^ range: 0.031-0.087; all *P_FDR_* < 0.05). After harmonization, the one-way ANOVA across scanners revealed no significant ROI-wise differences (all *P_FDR_* > 0.05). The partial η^2^ for the scanner factor was uniformly below 0.01 across all ROIs (range: 0.002-0.007), confirming that inter-scanner variability explained less than 1 % of the total variance and was therefore negligible.

**Supplementary Methods S3.** Detailed partial correlation procedure.

To eliminate the confounding effects of age, sex, years of education, and APOE ε4 carrier status, we employed the linear regression residual method. Specifically, for each pair of variables (X, Y), we regressed X onto the set of covariates and obtained the residuals; similarly, we regressed Y onto the same covariates and obtained its residuals. The Pearson correlation coefficient between the two residual sets was then taken as the partial correlation coefficient *r*. A total of 18 comparisons were tested, including structure-structure (HIP vs. CgH), structure-AD pathology (HIP/CgH vs. Aβ/P‑tau), and structure-cognition (HIP/CgH vs. Global cognition/Memory). The false discovery rate (FDR) procedure was applied to correct for multiple comparisons, with *P_FDR_* < 0.05 considered statistically significant.

**Supplementary Table S1.** Overall comparison statistics with effect sizes and 95% confidence intervals for demographic and neuropsychiatric variables among the five groups.

| **Variable** | **Statistic H/χ^2^** | **Effect Size V/ε^2^** | **95% CI** |
| --- | --- | --- | --- |
| Age | 15.254 | 0.067 | 0.016 - 0.176 |
| Gender | 0.963 | 0.075 | 0.059 - 0.268 |
| Education | 14.478 | 0.063 | 0.013 - 0.180 |
| APOE ԑ4 | 41.097 | 0.480 | 0.385 - 0.619 |
| MMSE | 93.660 | 0.537 | 0.467 - 0.629 |
| MoCA | 74.920 | 0.425 | 0.310 - 0.559 |
| WMS-LM | 116.95 | 0.676 | 0.605 - 0.741 |
| CDR | 151.437 | 0.883 | 0.826 - 0.930 |
| ADAS-Cog11 | 111.379 | 0.643 | 0.586 - 0.710 |
| ADAS-Cog13 | 110.481 | 0.638 | 0.574 - 0.713 |
| ADAS-Q4 | 94.223 | 0.540 | 0.451 - 0.652 |
| RAVLT immediate | 99.602 | 0.572 | 0.486 - 0.671 |
| RAVLT learning | 67.031 | 0.377 | 0.280 - 0.510 |
| RAVLT forgetting | 11.145 | 0.043 | 0.004 - 0.160 |
| RAVLT percent forgetting | 77.811 | 0.442 | 0.349 - 0.563 |

Continuous variables (non-normally distributed) were compared using the **Kruskal-Wallis H test**, with effect sizes quantified by **Epsilon-squared (ε^2^)**. Categorical variables (Gender and APOE ε4 status) were compared using the **Chi-square test**, with effect sizes quantified by **Cramer's V (V)**. CI = confidence interval; H = Kruskal-Wallis test statistic; χ^2^= Chi-square test statistic; ε^2^= Epsilon-squared effect size; V = Cramer’s V effect size

**Supplementary Table S2.** Partial correlations between MRI parameters, AD pathological proteins, and cognitive function (adjusted for age, sex, years of education, and APOE ε4)

|  | **Left** | | | | | **Right** | | | | |
| --- | --- | --- | --- | --- | --- | --- | --- | --- | --- | --- |
| **Variables** | ***r*** | **95% CI** | | ***P*** | ***P_FDR_*** | ***r*** | **95% CI** | | ***P*** | ***P_FDR_*** |
| HIP vs Aβ | 0.371 | 0.233 | 0.495 | <0.001 | <0.001 | 0.351 | 0.212 | 0.478 | <0.001 | <0.001 |
| HIP vs P-tau | -0.340 | -0.467 | -0.199 | <0.001 | <0.001 | -0.353 | -0.479 | -0.213 | <0.001 | <0.001 |
| CGH vs Aβ | 0.142 | 0.008 | 0.288 | 0.062 | 0.065 | 0.138 | -0.013 | 0.284 | 0.071 | 0.071 |
| CGH vs P-tau | -0.305 | -0.436 | -0.160 | <0.001 | <0.001 | -0.314 | -0.444 | -0.171 | <0.001 | <0.001 |
| HIP vs CGH | 0.232 | 0.083 | 0.370 | 0.002 | 0.003 | 0.224 | 0.075 | 0.363 | 0.003 | 0.004 |
| HIP vs Global Cognition | 0.412 | 0.278 | 0.530 | <0.001 | <0.001 | 0.373 | 0.235 | 0.497 | <0.001 | <0.001 |
| HIP vs Memory | 0.351 | 0.211 | 0.477 | <0.001 | <0.001 | 0.284 | 0.139 | 0.417 | <0.001 | <0.001 |
| CGH vs Global Cognition | 0.245 | 0.097 | 0.382 | 0.001 | 0.002 | 0.229 | 0.081 | 0.368 | 0.002 | 0.004 |
| CGH vs Memory | 0.211 | 0.062 | 0.351 | 0.005 | 0.006 | 0.191 | 0.041 | 0.333 | 0.012 | 0.014 |

All variables were residualized for age, sex, years of education, and APOE4 ε4 status. Significance level was set at α = 0.05 (two‑tailed). *r* = partial correlation coefficient; 95% CI = 95% confidence interval; *P* = uncorrected *P* value; *P_FDR_* = false discovery rate (FDR) adjusted *P* value. HIP = hippocampal volume; CGH = Hippocampal cingulum.

**Supplementary Table S3.** ANCOVA results for the Model 1 (without APOE ε4 adjustment) across 11 ROIs.

| **ROI** | **G1** | **G2** | **G3** | **G4** | **G5** | **F (4, df)** | ***P*** | **Partial η^2^**  **(95% CI)** |
| --- | --- | --- | --- | --- | --- | --- | --- | --- |
| L_HIP | 4.036 ± 0.077 | 3.718 ± 0.065 | 3.571 ± 0.082 | 3.332 ± 0.066 | 3.024 ± 0.065 | 28.082 | <0.001 | 0.398  (0.276, 0.480 ) |
| R_HIP | 3.986 ± 0.081 | 3.677 ± 0.069 | 3.579 ± 0.086 | 3.266 ± 0.069 | 2.909 ± 0.068 | 29.448 | <0.001 | 0.409  ( 0.288, 0.491 ) |
| L_PHG | 4.840 ± 0.093 | 4.663 ± 0.079 | 4.443 ± 0.099 | 4.283 ± 0.080 | 4.137 ± 0.079 | 10.577 | <0.001 | 0.199  (0.089, 0.286) |
| R_PHG | 4.852 ± 0.096 | 4.758 ± 0.082 | 4.455 ± 0.103 | 4.247 ± 0.083 | 4.066 ± 0.081 | 14.103 | <0.001 | 0.249  (0.131, 0.338) |
| L_AMYG | 1.352 ± 0.030 | 1.254 ± 0.026 | 1.203 ± 0.032 | 1.097 ± 0.026 | 0.980 ± 0.026 | 25.642 | <0.001 | 0.376  (0.253, 0.460 ) |
| R_AMYG | 1.307 ± 0.028 | 1.216 ± 0.024 | 1.133 ± 0.030 | 1.047 ± 0.024 | 0.966 ± 0.024 | 25.722 | <0.001 | 0.377  (0.254, 0.461) |
| L_CgH | 0.564 ± 0.008 | 0.543 ± 0.007 | 0.533 ± 0.008 | 0.507 ± 0.007 | 0.492 ± 0.007 | 15.24 | <0.001 | 0.264  (0.144, 0.352) |
| R_CgH | 0.554 ± 0.010 | 0.554 ± 0.009 | 0.514 ± 0.011 | 0.513 ± 0.009 | 0.485 ± 0.009 | 10.598 | <0.001 | 0.200  (0.089, 0.286 ) |
| GCC | 0.640 ± 0.008 | 0.635 ± 0.006 | 0.626 ± 0.008 | 0.608 ± 0.006 | 0.603 ± 0.006 | 5.562 | <0.001 | 0.116  (0.027, 0.193 ) |
| BCC | 0.602 ± 0.009 | 0.592 ± 0.008 | 0.587 ± 0.010 | 0.543 ± 0.008 | 0.515 ± 0.008 | 18.423 | <0.001 | 0.302  (0.180, 0.390 ) |
| SCC | 0.722 ± 0.008 | 0.708 ± 0.006 | 0.705 ± 0.008 | 0.669 ± 0.007 | 0.648 ± 0.006 | 18.544 | <0.001 | 0.304  (0.182, 0.392 ) |

All models were adjusted for age, sex, and years of education. Data are presented as estimated marginal means (Adj. Mean) ± standard error (SE), with F-statistics (degrees of freedom: 4 and residual df), P values, and partial η^2^ with 95% confidence intervals. G1 = A-T- CN, G2 = A+T- CN, G3 = A+T+ CN, G4 = A+T+ MCI, G5 = A+T+ AD. The results of inter-group multiple comparisons are shown in **Supplementary Figure S1.**

Abbreviations: CI, confidence interval; ROI, region of interest; HIP = Hippocampus; PHG = Parahippocampal gyrus; AMYG = Amygdala; CgH = Hippocampal cingulum; GCC = Genu of corpus callosum; BCC = Body of corpus callosum; SCC = Splenium of corpus callosum.

**Supplementary Table S4.** ANCOVA results for Model 2 (with APOE ε4 adjustment) across 11 ROIs, with Δη² compared to the Model 1.

| **ROI** | **G1** | **G2** | **G3** | **G4** | **G5** | **F (4, df)** | ***P*** | **Partial η^2^**  **(95% CI)** | **Δ η^2^** |
| --- | --- | --- | --- | --- | --- | --- | --- | --- | --- |
| L_HIP | 4.014 ±  0.08 | 3.705 ±  0.067 | 3.574 ± 0.082 | 3.349 ± 0.068 | 3.035 ± 0.066 | 23.812 | <0.001 | 0.365  (0.242, 0.452) | -0.033 |
| R_HIP | 3.987 ± 0.085 | 3.677 ± 0.07 | 3.579 ± 0.087 | 3.266 ± 0.072 | 2.909 ± 0.070 | 25.290 | <0.001 | 0.378  (0.254, 0.461) | -0.031 |
| L_PHG | 4.840 ± 0.097 | 4.663 ± 0.081 | 4.443 ± 0.100 | 4.283 ± 0.083 | 4.137 ± 0.080 | 8.782 | <0.001 | 0.172  (0.067, 0.257) | -0.027 |
| R_PHG | 4.888 ± 0.100 | 4.779 ± 0.084 | 4.450 ± 0.103 | 4.221 ± 0.085 | 4.048 ± 0.083 | 13.553 | <0.001 | 0.243  (0.125, 0.331) | -0.006 |
| L_AMYG | 1.354 ± 0.032 | 1.255 ± 0.026 | 1.203 ± 0.032 | 1.095 ± 0.027 | 0.979 ± 0.026 | 22.186 | <0.001 | 0.344  (0.221, 0.431) | -0.032 |
| R_AMYG | 1.305 ± 0.030 | 1.215 ± 0.025 | 1.133 ± 0.030 | 1.049 ± 0.025 | 0.967 ± 0.024 | 20.995 | <0.001 | 0.344  (0.220, 0.431) | -0.033 |
| L_CgH | 0.567 ± 0.008 | 0.545 ± 0.007 | 0.533 ± 0.008 | 0.504 ± 0.007 | 0.490 ± 0.007 | 14.862 | <0.001 | 0.260  (0.141, 0.349) | -0.004 |
| R_CgH | 0.558 ± 0.011 | 0.556 ± 0.009 | 0.513 ± 0.011 | 0.510 ± 0.009 | 0.483 ± 0.009 | 10.441 | 0.001 | 0.198  (0.087, 0.285) | -0.001 |
| GCC | 0.641 ± 0.008 | 0.636 ± 0.007 | 0.626 ± 0.008 | 0.607 ± 0.007 | 0.602 ± 0.006 | 5.084 | <0.001 | 0.107  (0.022, 0.183) | -0.008 |
| BCC | 0.600 ± 0.010 | 0.591 ± 0.008 | 0.588 ± 0.010 | 0.545 ± 0.008 | 0.516 ± 0.008 | 14.818 | <0.001 | 0.277  (0.156, 0.366) | -0.025 |
| SCC | 0.723 ± 0.008 | 0.708 ± 0.007 | 0.705 ± 0.008 | 0.668 ± 0.007 | 0.648 ± 0.007 | 16.411 | <0.001 | 0.280  (0.158, 0.368) | -0.024 |

All models were adjusted for age, sex, years of education and APOE ε4 status. Data are presented as estimated marginal means (Adj. Mean) ± standard error (SE), with F-statistics (degrees of freedom: 4 and residual df), P values, and partial η^2^ with 95% confidence intervals. Δη^2^ = partial η^2^ (Model 2) - partial η^2^ (Model 1). G1 = A-T- CN; G2 = A+T- CN; G3 = A+T+ CN; G4 = A+T+ MCI; G5 = A+T+ AD. The results of inter-group multiple comparisons are shown in **Supplementary Figure S2.**

Abbreviations: CI, confidence interval; ROI, region of interest; HIP = Hippocampus; PHG = Parahippocampal gyrus; AMYG = Amygdala; CgH = Hippocampal cingulum; GCC = Genu of corpus callosum; BCC = Body of corpus callosum; SCC = Splenium of corpus callosum.

**Supplementary Table S5.** Detailed effect sizes (Cohen’s d) and FDR-adjusted P values for APOE ε4 stratification within each subgroup across 11 ROIs.

| **Group** | **ROI** | **Cohen’s d** | **95% CI** | ***P*** | ***P_FDR_*** |
| --- | --- | --- | --- | --- | --- |
| G1 | L_HIP | -0.809 | (-1.974, 0.357) | 0.165 | 0.986 |
| G1 | R_HIP | 0.085 | (-1.081, 1.250) | 0.882 | 0.986 |
| G1 | L_PHG | 0.051 | (-1.114, 1.217) | 0.928 | 0.986 |
| G1 | R_PHG | 0.424 | (-0.742, 1.589) | 0.461 | 0.986 |
| G1 | L_AMYG | 0.113 | (-1.052, 1.278) | 0.843 | 0.986 |
| G1 | R_AMYG | 0.116 | (-1.049, 1.282) | 0.839 | 0.986 |
| G1 | L_CgH | 1.093 | (-0.073, 2.258) | 0.065 | 0.892 |
| G1 | R_CgH | 1.101 | (-0.065, 2.266) | 0.063 | 0.892 |
| G1 | GCC | 0.016 | (-1.149, 1.181) | 0.978 | 0.986 |
| G1 | BCC | -0.028 | (-1.194, 1.137) | 0.961 | 0.986 |
| G1 | SCC | 0.494 | (-0.672, 1.659) | 0.391 | 0.986 |
| G2 | L_HIP | 0.320 | (-0.353, 0.993) | 0.341 | 0.986 |
| G2 | R_HIP | -0.197 | (-0.870, 0.476) | 0.556 | 0.986 |
| G2 | L_PHG | -0.033 | (-0.706, 0.640) | 0.920 | 0.986 |
| G2 | R_PHG | 0.163 | (-0.510, 0.836) | 0.627 | 0.986 |
| G2 | L_AMYG | 0.274 | (-0.399, 0.947) | 0.415 | 0.986 |
| G2 | R_AMYG | 0.168 | (-0.506, 0.840) | 0.617 | 0.986 |
| G2 | L_CgH | 0.175 | (-0.498, 0.848) | 0.601 | 0.986 |
| G2 | R_CgH | 0.342 | (-0.332, 1.014) | 0.310 | 0.986 |
| G2 | GCC | -0.083 | (-0.756, 0.590) | 0.803 | 0.986 |
| G2 | BCC | -0.067 | (-0.740, 0.606) | 0.842 | 0.986 |
| G2 | SCC | -0.097 | (-0.770, 0.576) | 0.772 | 0.986 |
| G3 | L_HIP | -0.262 | (-1.302, 0.778) | 0.606 | 0.986 |
| G3 | R_HIP | -0.009 | (-1.049, 1.031) | 0.986 | 0.986 |
| G3 | L_PHG | -0.463 | (-1.503, 0.577) | 0.366 | 0.986 |
| G3 | R_PHG | -0.122 | (-1.161, 0.918) | 0.811 | 0.986 |
| G3 | L_AMYG | -0.172 | (-1.211, 0.868) | 0.735 | 0.986 |
| G3 | R_AMYG | 0.316 | (-0.724, 1.355) | 0.536 | 0.986 |
| G3 | L_CgH | 0.270 | (-0.770, 1.310) | 0.595 | 0.986 |
| G3 | R_CgH | -0.550 | (-1.589, 0.490) | 0.285 | 0.986 |
| G3 | GCC | 0.146 | (-0.894, 1.186) | 0.774 | 0.986 |
| G3 | BCC | 0.338 | (-0.702, 1.378) | 0.508 | 0.986 |
| G3 | SCC | 0.319 | (-0.721, 1.358) | 0.532 | 0.986 |
| G4 | L_HIP | -0.025 | (-0.899, 0.848) | 0.954 | 0.986 |
| G4 | R_HIP | 0.329 | (-0.544, 1.203) | 0.449 | 0.986 |
| G4 | L_PHG | 1.051 | (0.178, 1.924) | 0.020 | 0.892 |
| G4 | R_PHG | 0.952 | (0.079, 1.826) | 0.034 | 0.892 |
| G4 | L_AMYG | 0.148 | (-0.725, 1.022) | 0.732 | 0.986 |
| G4 | R_AMYG | 0.114 | (-0.760, 0.987) | 0.793 | 0.986 |
| G4 | L_CgH | 0.205 | (-0.668, 1.079) | 0.636 | 0.986 |
| G4 | R_CgH | 0.413 | (-0.461, 1.286) | 0.344 | 0.986 |
| G4 | GCC | 0.198 | (-0.676, 1.071) | 0.649 | 0.986 |
| G4 | BCC | -0.105 | (-0.978, 0.769) | 0.809 | 0.986 |
| G4 | SCC | 0.311 | (-0.562, 1.185) | 0.474 | 0.986 |
| G5 | L_HIP | -0.525 | (-1.367, 0.317) | 0.214 | 0.986 |
| G5 | R_HIP | -0.131 | (-0.973, 0.710) | 0.754 | 0.986 |
| G5 | L_PHG | -0.553 | (-1.394, 0.289) | 0.192 | 0.986 |
| G5 | R_PHG | -0.567 | (-1.409, 0.274) | 0.180 | 0.986 |
| G5 | L_AMYG | 0.035 | (-0.807, 0.876) | 0.934 | 0.986 |
| G5 | R_AMYG | -0.315 | (-1.157, 0.527) | 0.453 | 0.986 |
| G5 | L_CgH | -0.038 | (-0.880, 0.804) | 0.928 | 0.986 |
| G5 | R_CgH | -0.019 | (-0.861, 0.823) | 0.964 | 0.986 |
| G5 | GCC | 0.147 | (-0.695, 0.988) | 0.726 | 0.986 |
| G5 | BCC | -0.552 | (-1.394, 0.290) | 0.192 | 0.986 |
| G5 | SCC | -0.273 | (-1.115, 0.569) | 0.515 | 0.986 |

Data are presented as effect sizes (Cohen’s d) with 95% confidence intervals, nominal uncorrected *P* values, and FDR-adjusted q-values (*P_FDR_*) for comparisons between APOE ε4 carriers and non-carriers within each subgroup. Effect sizes and CIs were derived from ANCOVA models adjusted for age, sex, and education. FDR correction was applied using the Benjamini-Hochberg method across all 55 tests (11 ROIs × 5 subgroups). **All comparisons were non-significant after FDR correction (all *P_FDR_* > 0.05).** Although two comparisons (G4: L_PHG and R_PHG) showed nominal significance (nominal uncorrected *P* < 0.05), neither survived FDR correction (both *P_FDR_* = 0.892).

Abbreviations: CI, confidence interval; FDR = false discovery rate; ROI = region of interest; L = Left; R = Right; HIP = Hippocampus; PHG = Parahippocampal gyrus; AMYG = Amygdala; FFG = Fusiform gyrus; CgH = Hippocampal cingulum; GCC = Genu of corpus callosum; BCC = Body of corpus callosum; SCC = Splenium of corpus callosum.
